# Design, Implementation, and Evaluation of a Distance Learning Framework to Adapt to the Changing Landscape of Anatomy Instruction in Medical Education During COVID-19 Pandemic: A Proof-of-Concept Study

**DOI:** 10.3389/fpubh.2021.726814

**Published:** 2021-09-10

**Authors:** Nerissa Naidoo, Aida J. Azar, Amar Hassan Khamis, Mandana Gholami, Marjam Lindsbro, Alawi Alsheikh-Ali, Yajnavalka Banerjee

**Affiliations:** ^1^College of Medicine and Health Sciences, Mohammed Bin Rashid University of Medicine and Health Sciences (MBRU), Dubai, United Arab Emirates; ^2^Dubai Health Authority (DHA) Building, Dubai, United Arab Emirates; ^3^Centre for Medical Education, University of Dundee, Dundee, United Kingdom

**Keywords:** anatomy teaching, COVID-19, ADDIE model, Bourdieu's concept, competency framework, distance learning, instructional design and application, medical education

## Abstract

This study presents the design of a DL-framework to deliver anatomy teaching that provides a microfiche of the onsite anatomy learning experience during the mandated COVID-19 lockdown. First, using nominal-group technique, we identified the DL learning theories to be employed in blueprinting the DL-framework. Effectiveness of the designed DL-framework in anatomy teaching was demonstrated using the exemplar of the Head and Neck (H&N) course during COVID-19 lockdown, in the pre-clerkship curriculum at our medical school. The dissemination of the DL-framework in the anatomy course was informed by the Analyse, Design, Develop, Implement, and Evaluate (ADDIE) model. The efficiency of the DL-framework was evaluated using the first two levels of Kirkpatrick's model. Versatility of the DL-framework was demonstrated by aligning its precepts with individual domains of key learning outcomes framework. The framework's blueprint was designed amalgamating principles of: Garrison's community inquiry, Siemens' connectivism and Harasim's online-collaborative-learning; and improved using Anderson's DL-model. Following the implementation of the DL-framework in the H&N course informed by ADDIE, the framework's efficiency was evaluated. In total, 70% students responded to the survey assessing perception toward DL (Kirkpatrick's Level: 1). Descriptive analysis of the survey results showed that the DL-framework was positively received by students and attested that students had an enriched learning experience, which promoted collaborative-learning and student-autonomy. For, Kirkpatrick's Level: 2 i.e., cognitive development, we compared the summative assessment performance in the H&N course across three cohort of students. The results show that the scores of the cohort, which experienced the course entirely through DL modality was statistically higher (*P* < 0.01) than both the other cohorts, indicating that shift to DL did not have an adverse effect on students' learning. Using Bourdieu's Theory of Practice, we showed that the DL-framework is an efficient pedagogical approach, pertinent for medical schools to adopt; and is versatile as it attests to the key domains of students' learning outcomes in the different learning outcomes framework. To our knowledge this is the first-study of its kind where a rationale and theory-guided approach has been availed not only to blueprint a DL framework, but also to implement it in the MBBS curriculum.

## Introduction

In the past decade, medical schools around the globe have been endeavoring to renovate pedagogy by reducing didactic delivery of content; exploiting technology to supplant/augment anatomy teaching both during didactic and laboratory sessions; effecting team-expedited, active, and self-directed learning; and encouraging personalized and interprofessional education (1, 2). The expansion of entrustable professional activities (EPA) and competency-based learning, with recognized mileposts for accomplishment, have metamorphosed assessment. As a result of this so-called “renaissance in medical education,” several leading medical schools have reduced the basic science curriculum to 12 or 18 months, while amalgamating clinical medicine within this timeframe, and revisiting/reassessing the basic sciences later in medical school (3, 4).

At present, in most medical schools, students convene onsite during the first 12–18 months for collaborative problem-solving or discussions in small groups; their physical attendance at both inpatient and outpatient sites has been an undisputed precept of early clinical engagement experiences and the clerkship curriculum. The last 18 months of medical school may be customized, with students contributing to patient-care through advanced clinical rotations, sub internships preceding residency, or scholarly projects. The COVID-19 pandemic has affected this educational continuum.

Social distancing is the effectual preventive strategy since the emergence of COVID-19, pending completion of vaccination, treatment, or both (5, 6). By characterization, social distancing precludes students from gathering in learning ateliers, lecture halls, or small-group rooms. If one carefully follows the pedagogical trend in medical education over the past few years, one would observe that many faculty have already been “flipping” the classroom to provide individualized tutoring/instruction for asynchronous learning “anytime/anywhere” (7–9). However, students still convened for small-group interactions, laboratory sessions, simulations, and technology sessions [e.g., learning bedside medicine (10, 11)], as well as for clinical instruction with standardized patients and in realistic patient care environments (12).

In response to the pandemic, medical education faculty around the globe have rapidly and successfully transitioned the entire pre-clerkship curriculum to distance-learning (DL) formats that comprise content in the basic sciences, health systems sciences, and even in behavioral sciences (13–15). Small-group designs assemble online in virtual team settings (16), and clinical skills sessions may occur online employing the precepts of telemedicine (17) or, in some cases, may be deferred (18). Examinations have also migrated to online settings through the use of specific online proctoring modules (19). Even objectively structured practical examination (OSPE) have transitioned online in the form if electronic OSPE (20). Similarly, Web-Objective Structured Clinical Examination (OSCEs) have been piloted for the clinical skills assessment (21, 22). In summary, the pandemic has stimulated innovative, constructive and beneficial changes in medical education.

Anatomy education is an essential stipulation for medical students in the pre-clerkship curriculum. At heart, anatomy is a three-dimensional subject that necessitates understanding of body structures and their relationships. This aspect is often tackled using cadaveric specimens, access to which has been limited during the COVID-19 pandemic (23). Furthermore, organizing dissection of cadaveric specimens in small-group teaching milieus, often categorized as dissection sessions, have not been possible during the pandemic as the pre-clerkship curriculum delivery in most medical schools transitioned to the DL modality (24). In fact, this issue has added to the numerous challenges that are disadvantageously impacting anatomy education in current times as indicated above. These challenges include, a drastic reduction in anatomy teaching hours and its context, decrease in the number of trained anatomists, and an increase in the costs of human cadaveric dissections and the related ethical uncertainties surrounding the use of human cadavers (25). As there has been significant reduction in anatomy teaching hours, instructors often find it difficult to elaborate on the clinical relevancy of disseminated anatomical facts. This has initiated a new challenge in the form of “integration-gap” (26), whereby many students are of the opinion that learning anatomy largely involves rote-memorization as disseminated facts rarely inform their clinical practice in the clerkship years (27–29). Research by Patel and Moxham indicates that in order to address the aspect of “integration-gap,” majority of anatomists favor the use of human cadaveric dissection above other teaching methods, and that this preference is evident for both “traditionalist” and “modernist” anatomists (30, 31), which has also been confirmed by Ghosh (32). However, with the current pandemic, it is not possible to integrate onsite dissection sessions in anatomy teaching. Also, such onsite sessions are essential as such sessions not only provide students with haptic understanding and promote discussion of anatomical variation and pathologies, but also demonstrate aspects of professionalism, including empathy and respect for future patients. In fact, these relate directly to competency-based training in the surgery rotations of the clerkship curriculum, which rely on defined EPAs to assess and document competence (33). One of the alternate strategies to address ‘integration gap’ is to integrate simulation-based anatomy teaching (34), however, this pedagogical approach also demands onsite sessions in the in the simulation laboratory, not possible during the current pandemic.

In summary, keeping in line with COVID-forced challenges, the landscape of anatomy teaching needs to metamorphose, whereby there is an ardent requirement to design a “student-centered teaching framework” (easily implementable for both face-to-face and DL modalities), such that anatomy can be delivered effectively: (1) within a limited and fixed time frame [in line with recent the transformations in pre-clerkship curriculum (stated above)]; (2) employing a small team of trained anatomists; (3) using a small number of cadaveric specimens; (4) by integrating principles of active learning, collaborative learning, feedback, and student autonomy; and (5) in a cost-effective approach.

Although, the literature provides a corpus of information on DL strategies that have been designed and implemented during the COVID-19 pandemic for the delivery of courses in the pre-clerkship phase of the curriculum, none of these strategies provide a theory-guided approach, such that these DL strategies can be adopted for the delivery of any course in a competency based medical curriculum. Moreover, most of the reported DL strategies rarely integrate an instructional design model to facilitate the delivery of a course. The purpose of this study is to address these gaps. Therefore, in this study, we have designed a DL-framework to deliver anatomy teaching that provides a microfiche of the onsite anatomy learning experience during the mandated COVID-19 lockdown. First, through a dedicated needs-assessment using nominal-group technique (35), we identified the DL learning theories that should be employed in blueprinting the DL-framework. The framework's blueprint was designed amalgamating principles of:

Garrison's community inquiry (36, 37), Siemens' connectivism (38) and Harasim's online-collaborative-learning (39); and improved using Anderson's DL-model (40). Effectiveness of the DL-framework in anatomy teaching and course delivery was demonstrated using the exemplar of the Head and Neck (H&N) course during the COVID-19 lockdown in the pre-clerkship phase of the competency-based medical curriculum (CBMC) at our medical school. Instructional strategy integrated in the H&N course to disseminate the DL-framework were informed by the Analyse, Design, Develop, Implement, and Evaluate (ADDIE) instructional design model (41). The efficiency of the DL-framework was evaluated using the first two levels of Kirkpatrick's model (42) as part of ADDIE. Additionally, using Bourdieu's Theory of Practice (43), we demonstrate that the DL-framework is an efficient pedagogical approach, pertinent for medical schools to adopt; and is robust and versatile, as it attests to the key domains of different learning outcomes framework. In summary, the strategized DL-framework presents an adaptable approach for medical education faculty to efficiently and effectively deliver anatomy courses in the pre-clerkship curriculum in the changing landscape of anatomy instruction, especially during unprecedented circumstances as presented by the COVID-19 pandemic.

## Methods

### Study Landscape

Mohammed Bin Rashid University of Medicine and Health Science (MBRU) is medical school located in Dubai, where the curriculum is founded on a competency-based educational model (44), in line with the tenets of Epstein and Hundert (45), and spans over 6 years. The undergraduate entry medical curriculum provides a milieu for a highly adaptive learning process rather than the traditional “one-size-fits-all curriculum.” Furthermore, the MBRU curriculum aims to foster an erudition environment, where peer-assisted learning and learning supported by social learning theories are facilitated (46).

**Figure 1:**
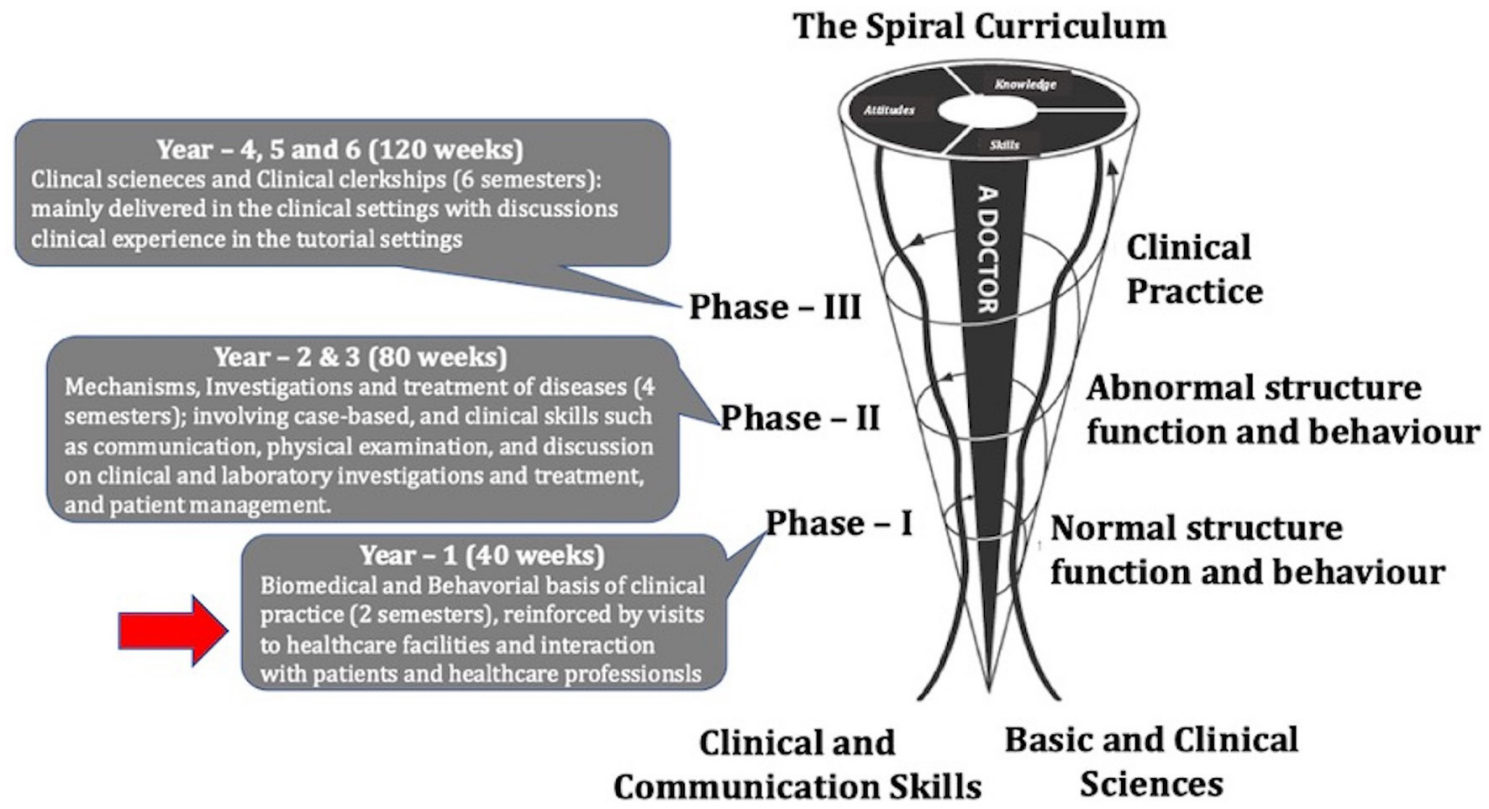
The undergraduate medical curriculum at Mohammed Bin Rashid University of Medicine and Health Sciences (MBRU). The curriculum is divided into three phases and spans over 6 years. Note: Each phase of the undergraduate medical curriculum includes integrated courses and builds on the preceding one, such that the curriculum is a “spiral,” and the students repeat the study of a subject, each time at a higher level of difficulty and in greater depth. The phase in which the teaching framework was implemented is indicated with a red arrow.

The MBRU curriculum is divided into 3 phases (Figure 1). The pre-clerkship curriculum is divided into two phases: Phase -I and Phase -II and spans over 3 years. The clerkship curriculum consists of Phase–III courses, which focus on clinical training. Each phase of the curriculum includes integrated courses and builds on the preceding one, such that the curriculum is “spiral” and the students repeat concepts pertaining to a subject, where with each successive encounter, concepts build on the previous one (47) (Figure 1).

### Participants

The study participant were selected according to a defined inclusion criteria. Only students in the second semester of Phase – I (Figure 1), registered for the H&N anatomy course were eligible to participate in the study. A total of 56 students who had successfully completed all the courses in semester – 1 of Phase – I registered for the course.

Participation in this study was entirely voluntary. Of the total 56 students registered, only 39 (70%) students opted to participate in this study. Gender distribution was represented by 26 (67%) females and 13 (33%) males, with the female to male respondent ratio representative of that of the entire cohort. The mean age of the study participants was 19.2 (3.2) years.

### Dissemination of the DL Framework

The incorporation of the ADDIE Model (41) (refer below for details) enabled us to implement the pedagogical approach of flipped learning in the DL delivery of the H&N course. The cloud-based software, Brightspace learning management system (LMS) by D2L (Kitchener, ON, Canada) was employed for dissemination of the course content, and formative assessments.

Instructional materials in the form of the study-guide, pre-recorded PowerPoint presentations [which an initial survey indicated was the mode of content delivery preferred by the students (*data not shown*)], data sets and clinical cases to be discussed and formative assessments were uploaded on the LMS by the concerned instructor(s) at least 1-week prior to the delivery of a given session. The LMS was linked to an intelligent timetabling module by Wise Technologies Ltd (Ljubljana, Slovenia), which allowed students to simultaneously view the weekly schedule of the course-sessions with the intended learning outcomes for each session.

Due to the mandated COVID-19 lockdown and social-distancing, all on-campus sessions were substituted with virtual off-campus live sessions, organized using Microsoft (MS) Teams (MS Corporation, USA) application compatible with Windows, Linux, macOS, iOS and Android operating systems. Prior to each live session, a reminder email was sent to all students registered for the course to ensure their participation. Discussion groups were conducted using WhatsApp (Facebook, Inc.).

### Design of the Framework for Course Dissemination

#### Identifying the Learning Theories for the Framework Using Nominal Group Technique

**Figure 2:**
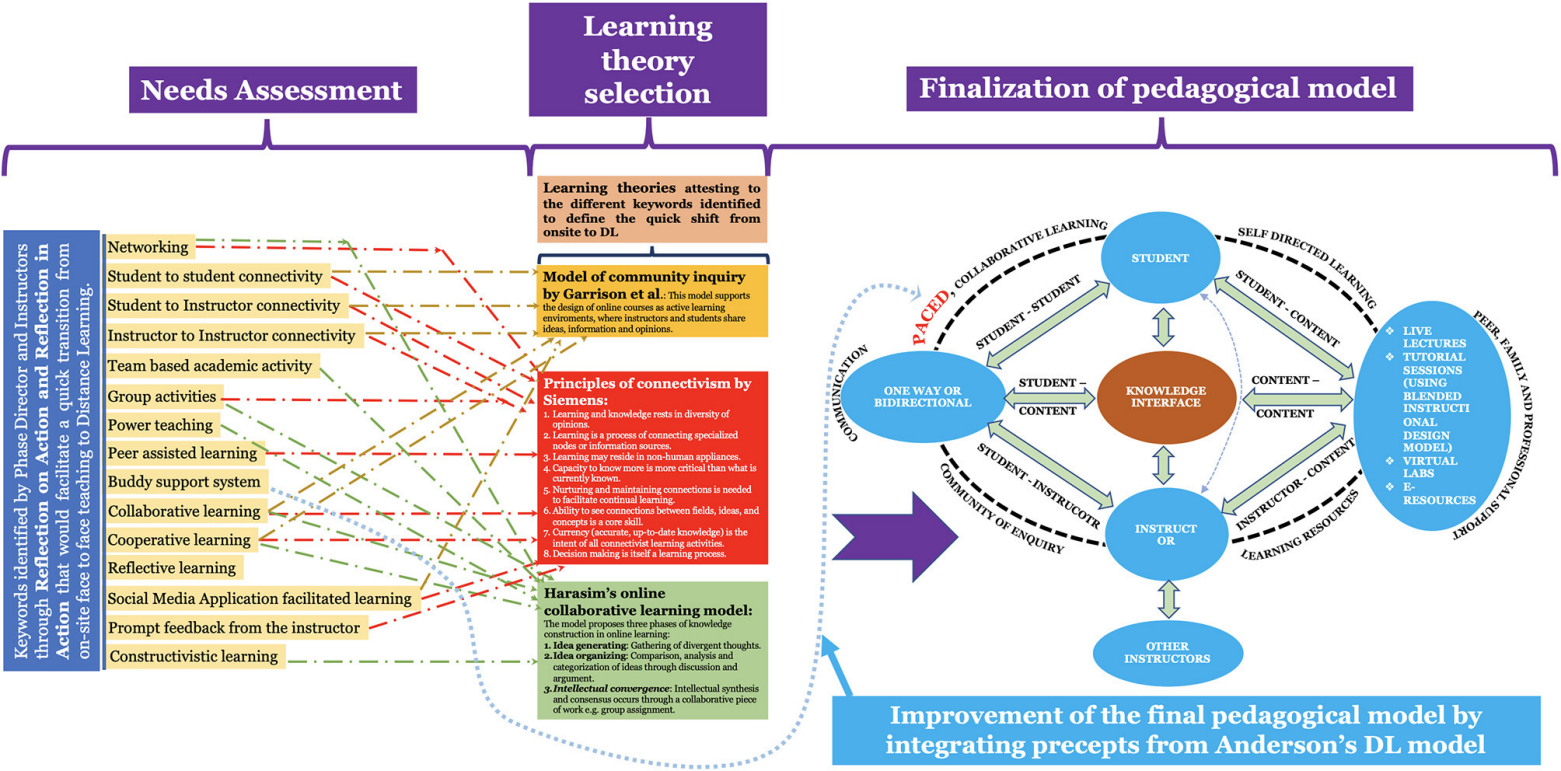
Identification of the DL theories using nominal group technique, and their use in blueprinting the DL-framework. The framework's blueprint was designed amalgamating principles of: Garrison's community inquiry, Siemens' connectivism and Harasim's online-collaborative-learning; and improved using Anderson's DL-model. Note: The need for the incorporation of the “Buddy support system” required us to integrate the Anderson's DL-model (refer to the text for details).

The blueprint of the pedagogical framework was designed amalgamating principles of: Garrison's community inquiry (36), Siemens' connectivism (38) and Harasim's online-collaborative-learning (39); and improved using Anderson's DL-model (40) (Figure 2). These learning theories were identified using keywords collated employing nominal group technique (48) (Figure 2). Briefly,

- In the *Orientation phase* (~10 min), the director of Phase – I of the curriculum along with the course coordinator and lead instructor of the H&N course formed a team; where the Phase – I director informed the team members to individually identify keywords using principles of Schon's “Reflection in action and Reflection on Action model” (49, 50); that they believed best attested to the “learning needs” of the students and would facilitate a rapid transition of the H&N course from on-site face-to-face teaching to the DL modality. A list of questions, identified by Menard et al. (51), was also circulated among the team members to trigger guided reflection.
- Team members wrote their responses on 3 in × 5 in post-it notes independently and silently (*Idea generation phase*), following which each member read their responses to others in the team *(Idea sharing phase*). The total process took ~15 min.
- In the *Group discussion phase* (~25 min), members explained to the team the rationale for selecting a specific keyword. A final list of keywords was collated, and any duplicates were removed.
- In the *Voting and Ranking phase* (~10 min), the team members voted on the ranking of keywords according to their importance.

#### Selection of the Learning Theories

The Phase – I director and the course coordinator used the ranked keywords to search for learning theories. Four education-relevant databases: ERIC (https://eric.ed.gov); PubMed (https://pubmed.ncbi.nlm.nih.gov); PsychINFO (https://search.proquest.com/psycinfo/advanced); and Web of Science (https://webofknowledge.com) were searched for publications associated with DL theories using the list of ranked keywords obtained using nominal group technique (Figure 2).

**Figure 3:**
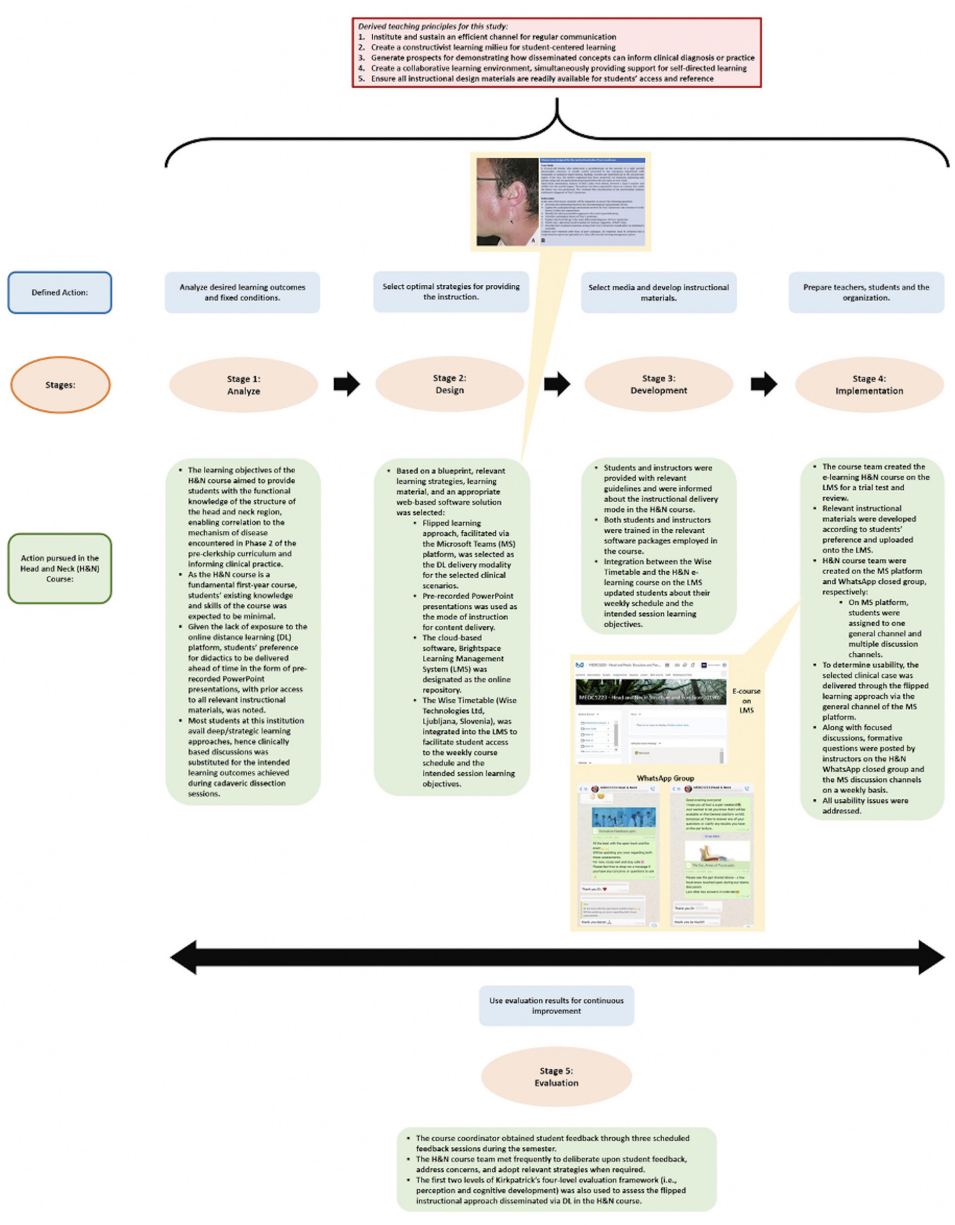
Defined activities pertaining to each stage of the ADDIE instructional design model through which the flipped classroom approach was delivered in the H&N course during the mandated COVID−19 period, where the designed DL pedagogical strategy was implemented. Note: the flipped learning approach was employed in the instruction of selected clinical cases (shown using the exemplar of Frey's Syndrome in figure) that was delivered through the general channel of the MS teams platform. Formative questions and interesting facts pertaining to the selected clinical cases were posted weekly by instructors on the H&N WhatsApp closed group (snippets of this shown in the figure) and the MS teams discussion channels, allowing students to self-evaluate their understanding of the delivered concepts and establish a community of learning through focused discussions.

Three DL theories were identified from 21 publications, which were later vetted by the team for eligibility: Garrison's community inquiry (36), Siemens' connectivism (38), and Harasim's online-collaborative-learning (39) (Refer to Figure 2 for the key principles of individual theory). These three DL theories were used to construct the initial pedagogical model. However, in order to integrate the aspect of “Buddy support system” (Figure 2), which essentially focuses on collaborative learning (52), we decided to integrate principles of Anderson's DL-model (40), to obtain the final pedagogical model (Figure 2). The final pedagogical model was vetted and reviewed by the team. This model was used to derive the teaching principles. The teaching principles were further employed to blue-print the guide-plan for the DL course (Figure 3). Based on the derived teaching principles, there was a consensus to adopt ADDIE (Analysis, Design, Development, Implementation, and Evaluation) (41) to deliver the H&N course (Figure 3).

### Rationale Behind the Selection of the ADDIE Model to Design the Dissemination Framework for the Course

One of the essential aspects to be considered when designing a framework for the dissemination of a course is that the instructors must ensure that the framework provides a coherent and favorable learning milieu for all students. The ADDIE model is one such framework, and has been used to design and blueprint curriculum delivery in diverse fields such as online continuing education (53).

Also, in light of the unprecedented circumstances, brought about by the COVID-19 pandemic, as well as the evolving response in governmental and institutional measures, it was important to adopt an instructional model which offers instructors considerable flexibility. ADDIE's inherent characteristics of flexibility and non-linearity allow instructors to easily adapt and combine it, and the information and elements can be adjusted and modified to meet the needs of the course and its learning objectives (54). Based on the above, we decided to incorporate the ADDIE model in the instructional delivery of the H&N course.

Furthermore, the ADDIE model can be rapidly implemented in the delivery of a course due to its simplicity (55). This is evident from the fact that the model has been extensively used by the United States army, especially during World War II, when the armed forces had to swiftly conduct technical training for a large group of new recruits who were expected to uphold various specialized military roles (56). Our situation was somewhat similar. We had little time to transition from onsite to the DL modality, and also our delivery strategy required to facilitate the integration between basic science and clinical concepts. To address both these requirements the ADDIE model was considered to be best-suited for designing the framework for the delivery of the H&N course (57).

### Blueprint of the Course Dissemination Framework Designed Using ADDIE

#### Stage 1: Analysis

This stage was considered to be integral to the framework as it inadvertently posed questions that acted as an investigative prelude to the implementation process (54). Thus, through this stage, the instructional problem was elucidated, instructional goals and objectives were established, and the students' learning environment, existing knowledge, and skills were ascertained (54). The H&N course team and the Phase I Director, engaged in a discussion to identify the instructional challenges associated with course delivery in the context of setting and modality and determined the expectations for performance after course completion (Figure 3).

As a fundamental 1st-year course, the H&N course is designed to provide students with the functional knowledge of the structure of the head and neck region, thereby enabling correlation to the mechanism of disease encountered in Phase 2 of the pre-clerkship curricular phase, so as to inform clinical practice. In this regard, it was expected that students' existing knowledge and skills of the H&N course would be minimal.

In view of the learning context and target group, institutional instructional delivery was transferred to the online DL platform due to the mandated COVID-19 lockdown. Since this particular student cohort did not have previous exposure to the DL modality, an informal survey, enquiring about preferred mode of DL delivery, was conducted to address the transition to the DL platform. The majority of students indicated that they preferred the didactics to be delivered ahead of time in the form of pre-recorded PowerPoint presentations (*data not shown*), with prior access to all relevant instructional materials. Moreover, one of our earlier studies, indicated that most students at MBRU avail deep/strategic learning approaches. Therefore, we decided to incorporate “*clinical case discussions*” as a substitute for the intended learning outcomes achieved during *cadaveric dissection sessions* (25, 58). Further, it was decided that precepts pertaining to aspects of “Balint groups” (59) and “Schwartz rounds” (60) will be integrated in the clinical case discussions to promote collaborative learning and discussions.

#### Stage 2: Design

This phase entailed the creation of a blueprint which was based on the feedback generated from the discussion of the analysis stage (54). This led to the selection of relevant learning strategies, learning material, and a web-based software solution to support the instructional process (Figure 3).

Accordingly, the flipped learning approach, facilitated via the MS Teams platform was selected as the DL strategy to deliver the dedicated selected clinical case discussions. An exemplar of a clinical case pertaining to Frey's Syndrome is shown in Figure 3. LMS was designated as the online repository through which students accessed instructional material. In addition, integration of the scheduling assistant, Wise Timetable, assisted students to keep a record of their learning progress.

#### Stage 3: Development

In this phase, the course team evaluated the designed material (54) (Figure 3). In accordance with the blueprint, the course team created the e-learning H&N course on the LMS for a trial test and review. The relevant instructional materials, developed in line with students' preference as stated in Stage 1, were uploaded onto the LMS.

A H&N course team with one general channel and multiple discussion channels were created on the MS Teams platform. The entire student cohort, assigned to the general channel on MS Teams platform, was then divided into two large groups, each of which were further sub-divided into smaller teams and assigned to the respective discussion channels for the purpose of clinical case discussions.

In effort to determine usability in the initial demonstration phase, the flipped learning approach was employed in the instruction of the selected clinical case that was delivered through the general channel of the MS Teams platform. After a thorough discussion with the course team, a H&N WhatsApp closed group was created. Formative questions and interesting facts pertaining to the selected clinical cases were posted weekly by instructors on the H&N WhatsApp group and the MS Teams discussion channels, allowing students to self-evaluate their understanding of the delivered concepts and establish a community of learning through focused discussions (Figure 3).

Once all usability issues were addressed, the instructional design framework progressed to Stage 4 (i.e., implementation) (Figure 3).

#### Stage 4: Implementation

This phase entailed the preparation of students and instructors by providing relevant guidelines and informing them about the particulars pertaining to the instructional delivery mode in the H&N course (Figure 3). Both students and instructors were trained in the relevant software packages employed in the course. Integration between the Wise Timetable and the H&N e-learning course on the LMS ensured that the students were regularly informed about their weekly schedule and the intended session learning objectives.

#### Stage 5: Evaluation

This phase ensured that the H&N course underwent continuous improvement as the course progressed through the semester (Figure 3). Accordingly, student feedback was obtained thrice during the course through scheduled feedback sessions organized by the course coordinator. The H&N course team, which was chaired by the course coordinator, met frequently to deliberate upon this student feedback, address concerns, and adopt relevant strategies when required. In addition, the flipped instructional approach disseminated via DL, was also assessed using the first two levels of Kirkpatrick's four-level evaluation framework (42), i.e., perception and cognitive development (*Refer below for details*).

### Evaluation of the DL Framework

DL in the H&N course was evaluated using the first two levels of Kirkpatrick's four-level evaluation framework (42). Level – 1 of the framework evaluates reaction/perception, i.e., “*Did the student/learners enjoy the learning process?*” Level – 2 of Kirkpatrick's framework evaluates cognitive development, i.e., “*Did the learning occur?*”

#### Kirkpatrick's Level 1 Evaluation

(a) Survey tool: A 19-item 5-point Likert scale (1 = strongly disagree; through 5 = strongly agree) survey, adopted from one of our previous studies with minor modifications (61), was used to evaluate Kirkpatrick's Level 1 (*Refer to supplementary information for the questionnaire*) i.e., students' perception regarding the DL delivery of the H&N course during the mandated COVID-19 pandemic lockdown. As this was the first time the students experienced anatomy instruction through the DL modality, we perceived that it was imperative to appraise “Anxiety associated with the use of DL” (one question item) (*Refer to supplementary information for details*). The remaining 18-question items were further grouped into four principal categories: *Computer expertise* (four question items); *Flexibility of DL* (four question items); *Usefulness of DL* (five question items); and *DL Satisfaction* (five question items) (*Refer to supplementary information for details*).
(b) Validation of the survey: The survey tool was assessed for both validity and reliability. The statistical package, SPSS (V25, IBM Corp, NY, USA) was used to analyze the data.

According to Tabachnick and Fidell (62), sample sizes of 50 and 100 are considered to range between very poor and poor. As we had 56 participants in this study, we decided to employ the Kaiser-Meyer-Olkin (KMO) test to measure sample adequacy (MSA) (63). The KMO test, which correlates pairs of variables and the magnitude of the partial correlations among variables, ideally yields an overall measure within the range of 0.6 and 1. A high (1.0) KMO index indicates that the Principal Components Analysis may be conducted, while a low (~0.0) KMO index states otherwise (63).

Intercorrelations between variables (i.e., interrelation of each question item in a specific category) were determined through the statistical method of exploratory factor analysis (EFA) (64) and, the Bartlett test of sphericity (65).

EFA explores previously unknown groupings of variables to seek underlying patterns, clustering and groups. Loading values below 0.5 indicate that the specific item has a weak influence on that category, values between 0.5 and 0.6 indicate that the specific item has a fair influence, values between 0.6 and 0.7 have a sufficient factor loading influence, values between 0.7 and 0.8 have a good loading influence, and those above 0.8 have a high loading influence on that category (66).

The Bartlett test, which investigates the correlations between variables, should reveal statistical significance (*P* < 0.05) (especially used when the number of cases per variable is five or fewer).

The internal consistency (reliability) of the questionnaire was validated using the Cronbach's alpha (α). This measures the extent to which the question items within each category consistently measure what is intended to be measured. Values of α below 0.6 indicate low internal consistency, values between 0.6. and 0.7 indicate acceptable consistency levels, good levels are those values between 0.7 and 0.8, and high reliability levels are those above 0.8 (67).

#### Kirkpatrick's Level 2 Evaluation

Level 2 of Kirkpatrick's framework evaluated students' cognitive development in the H&N course. This was addressed by comparing the summative assessment performance across three cohort of students.

Cohort 1 – experienced the course through traditional teaching modality (with onsite teaching and dissection sessions) without the presence of a pedagogical framework; Cohort 2 – experienced the course through traditional teaching modality with presence of a defined instructional design framework, which was applied in a blended learning milieu in one of our earlier studies (25), and Cohort 3 (*current cohort*) – experienced the course entirely through DL modality.

**Figure 4:**
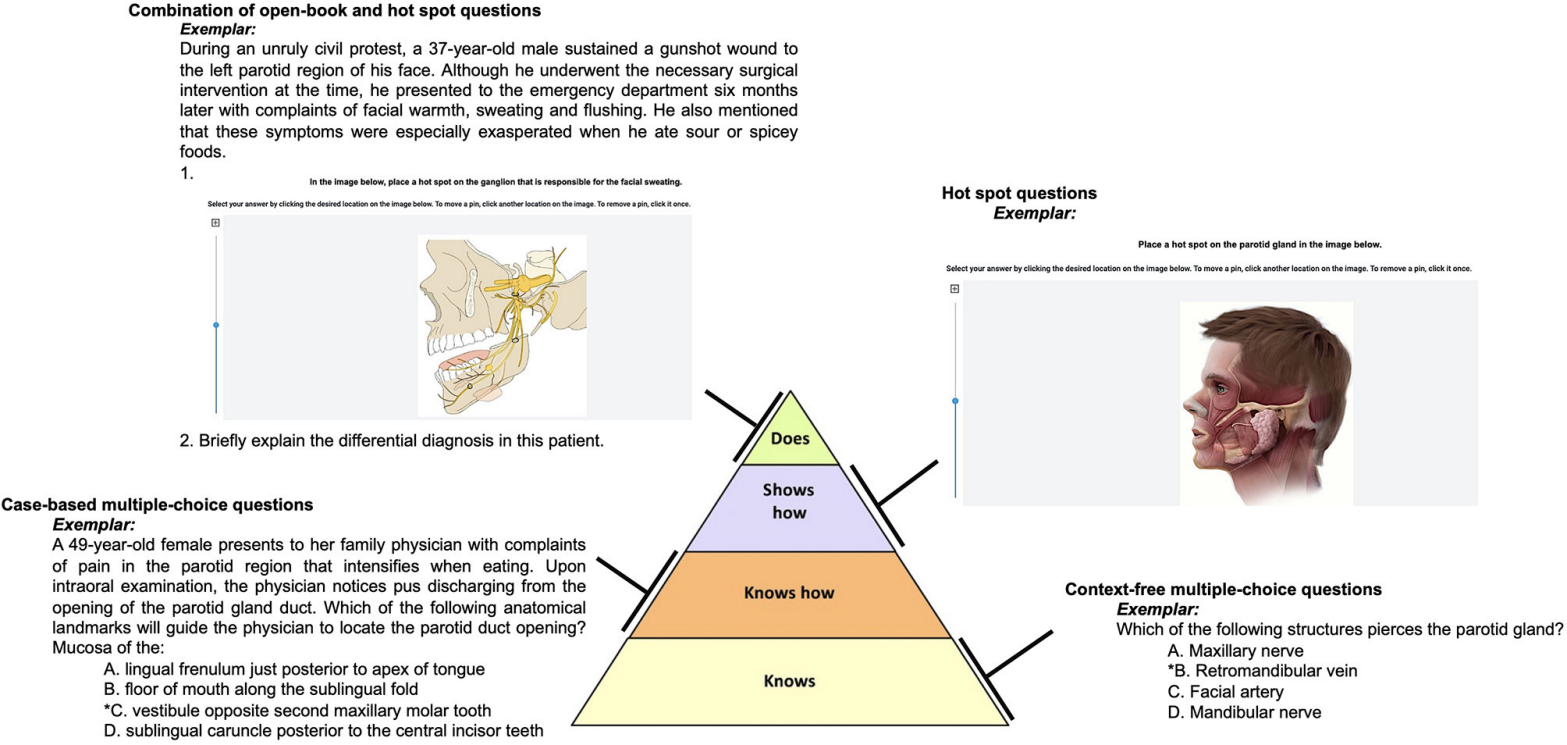
Design of summative assessment. Summative assessment was designed according to the principles of Miller's pyramid of professional competence integrating different assessment tools.

The summative assessment content and structure was similar for all the three cohorts, and was standard set by a panel of content experts assigned by the student assessment and progression committee (SAPC) at our medical school. The summative assessment was designed according to the principles of Miller's pyramid of professional competence (68) and employed different types of assessment tools, which is summarized in Figure 4. However, we could not include practical assessment in the cohort which was exposed to the DL-framework (Cohort 3), due to mandated social distancing protocols in line with the pandemic. The summative assessment scores across the different cohorts (indicated above) was compared using the Analysis of Variance (ANOVA) (69). The software package of Stata (StataCorp V16, CA, USA) was used for this analysis. A *p* < 0.05 was considered statistically significant (70).

## Results

### Validation of the Survey Tool

In this study, the validity and reliability of the questionnaire is shown in Table 1.

**Table 1:** Validity and reliability analyses of the questionnaire administered to 1st-year medical students (*N* = 39) undertaking the Head and Neck Course during the mandated COVID-19 lockdown^a^.

**Category**	**Items in each category**	**Exploratory factor analysis**		**KMO^b^ & Bartlett**	**Cronbach's Alpha^c^ (%)**
		**Factor loading value**	**Item influence**		
Computer expertise	This course helps me use the internet source more efficiently	0.68	Sufficient	0.70^d^	77.9
	My use of computers increases after taking this course	0.74	Good		
	My computer knowledge increases with the course assignments and projects	0.81	High		
	This course contributes to my knowledge of searching on the internet	0.89			
Flexibility of distance learning	In terms of use of time and location, distance learning is flexible	0.63	Sufficient	0.54^d^	73.0
	Distance learning is appropriate to students with different learning capacities	0.66			
	Distance learning allows me to allocate my time better	0.76	Good		
	Distance learning allows me to work at home comfortably	0.93	High		
Usefulness of distance learning	Evaluation of the success in distance learning is quite objective	0.48	Weak	0.73^d^	77.4
	Distance learning provides me with a valuable learning experience	0.76	Good		
	A degree in distance learning is as valuable as a degree in traditional education	0.79			
	I believe distance learning is useful	0.83	High		
	Distance learning minimizes the inequalities in education	0.85			
Distance learning satisfaction	In this course, I am pleased with the timely responses to my questions	0.57	Fair	0.75^d^	68.1
	The content of this course meets my expectations	0.61	Sufficient		
	I advise other students to take this course	0.63			
	I like the content of the course which draws examples from real life	0.65			
	The student-centred instruction offered in this course through distance learning is enjoyable	0.92	High		

a *First-year medical students enrolled in the Bachelor of Medicine, Bachelor of Surgery (MBBS), College of Medicine, Mohammed Bin Rashid University of Medicine and Health Sciences (MBRU), Dubai, United Arab Emirates*.

b *KMO, Kaiser-Meyer-Olkin*.

c *Shows the internal consistency*.

d *P < 0.0001*.

Sample adequacy was confirmed by KMO values yielded within the range of 0.6 and 1.0 for categories “Computer expertise,” “Usefulness of distance learning” and “Distance learning satisfaction” (Table 1). However, the category “Flexibility of distance learning” yielded a KMO value of 0.54, which may be attributed to the low sample size that was restricted to only 56 students within the cohort who registered for the H&N course (Table 1). This category was not removed from the analysis as the KMO value is close to 0.6. More relevantly, the Bartlett test was statistically significant for all categories, revealing a strong correlation between the items in each category (Table 1).

As indicated by the EFA values (0.60–1.0), most question items (i.e., 16 items) were noted to have either a sufficient, good or high influence on their respective categories (Table 1). Two questionnaire items emanating from the categories “Usefulness of distance learning” and “Distance learning satisfaction” yielded EFA values that were <0.40, indicating weak and fair influences, respectively (Table 1). Cronbach Alpha values were >0.6 for all categories in this study, revealing acceptable to good levels of internal consistency between question items, thereby validating the reliability of the questionnaire employed (Table 1).

### Kirkpatrick's Level 1 Evaluation: Evaluation of a Student's Perception

Level 1 of Kirkpatrick's framework (i.e., perception) was evaluated using a validated 18-item 5-point Likert scale questionnaire, grouped into four categories (*Refer to methods for details*) preceded by an item appraising the association of anxiety with the use of DL. The results obtained with students' perception are presented and discussed below in detail.

#### Anxiety and the Use of Distance Learning (DL)

In the survey tool, one of the items appraised student anxiety associated with the use of DL. This item specifically enquired about the anxiety associated with the transition of onsite face-to-face teaching to the DL modality.

**Figure 5:**
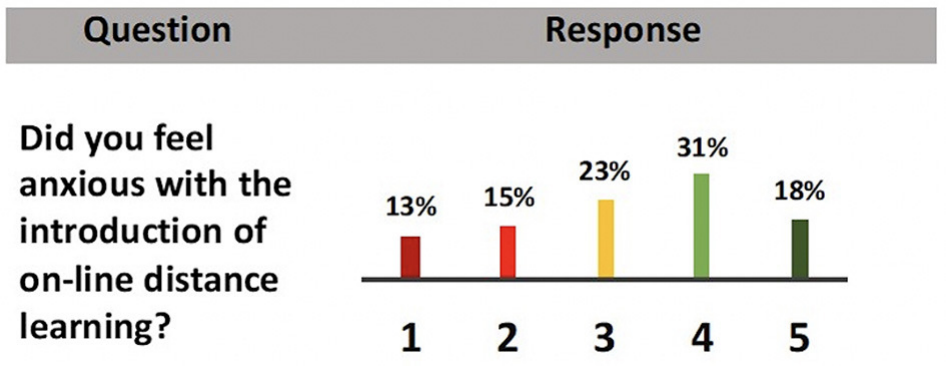
Bar graph showing respondents' levels of agreement about anxiety and the use of DL in the H&N course following the implementation of the blueprinted DL framework. (5-point Likert scale: 1 = strongly disagree through 5 = strongly agree) (refer to the text for details).

With the precipitous shift from onsite face-to-face teaching to DL, majority (49%) of students expressed that they felt anxious (Figure 5). Anxiety is a fundamental human emotion that consists of fear and indecision and typically occurs when an individual believes that the event is a threat to self or self-esteem. Findings suggest that students, as well as the general population, may be experiencing negative psychological effects from the outbreak of COVID-19, such as anxiety, fear, and worry, among others (71). Also, students feel more anxiety in DL due to the distance as they are unable to discuss or share problems with instructors on a daily basis (72). Additionally, collaborative learning associated with DL often becomes difficult giving rise to anxiety (73).

For the instructor, the indication of understanding, or lack thereof, that is observed from the general facial expressions and body language of students in the class, is also absent, thereby requiring students to vocalize their feedback via the DL platform, which further adds to the anxiety as students are often shy to voice their opinions and feedback (74). Moreover, students with socially anxious tendencies may not be comfortable to approach instructors for clarification on content, creating “gap in knowledge,” which may further add to the anxiety of students experiencing a course through DL. This aspect is supported by the observed direct proportionality reported between academic underperformance and academic anxiety.

To resolve these concerns, it is recommended that positive instructor-student relationships be developed along with the integration of regular instructor feedback and counseling initiatives into the DL approach, thus helping students adapt to this new modality. In fact, at MBRU we have initiated sessions involving progressive muscle relaxation (PMR) to counter anxiety in students pursuing medical education [Naidoo et al., manuscript under consideration (refer to supplementary information for the manuscript file)].

It is to be noted that 28% of the students conveyed that they did not experience emotions of anxiety with the transition to DL (Figure 5). This may be attributed to the fact that MBRU draws medical students from diverse high school curricula locally and internationally, coming from different socio-economic standings and perhaps with previous exposure to the DL modality. It is also likely that the respondent group is representative of adult learners, who were able to quickly adapt to the transition to DL (75).

Anxiety also has direct bearing on the mental health and learning ability of a medical student (76–78). However, one key aspect of anxiety often not elaborated upon in the medical education literature is that it also has a genetic predisposition (79). This issue of genetic predisposition is important in the current study, given the fact that MBRU medical student population is multi-ethnic, which alludes to the fact that different genetic makeup may be an underlying factor when it comes to the aspect of coping with anxiety related stress. In a study by Hill et al., a genome-wide analysis study (GWAS) showed the direct influence of 30 different loci on socio-economic status of an individual; indicating the effect of environmental and genetic factors on brain structure and neuronal function, which in turn affects education, intelligence, income and the ability to handle stress (80). This is also supported by the studies of Zhou et al., where genetic variation in human neuropeptide Y expression affects psychobiology and molecular aspects of stress response and resilience (81, 82). In line, the observed difference in the ability to handle stress may have a underlying genetic causality, which of course needs to be explored further using a relatively genetically homogenous population of students, as available in some of the countries in the region (83).

#### Computer Expertise and the Use of Distance Learning

Category 1 or Computer Expertise comprised of four items that focused primarily on the effect of DL on computer expertise. Computer expertise can be defined “*as the abilities of the students to work efficiently with a computer*.” For the first three items under this category, 59–72% of responses were in the agree-strongly agree continuum, with 8–11% of responses ranging between strongly disagree and disagree (Table 2). Majority of responses within the agree-strongly agree continuum, indicates that the DL modality augmented the computer expertise of the course participants. We believe this has long-term benefits.

**Table 2:** Levels of agreement for different categories.

		**Likert scale, levels of agreement in %**				
**Category**	**Item**	**SD**	**D**	**N**	**A**	**SA**
Computer expertise	This course helps me use the internet source more efficiently	0	8	28	59	5
	My use of computers increases after taking this course	3	8	18	36	36
	My computer knowledge increases with the course assignments and projects	3	18	33	46	0
	This course contributes to my knowledge of searching on the internet	0	8	33	49	10
Flexibility of distance learning	In terms of use of time and location, distance learning is flexible	0	0	87	10	3
	Distance learning is appropriate to students with different learning capacities	3	33	23	26	15
	Distance learning allows me to allocate my time better	18	18	18	33	13
	Distance learning allows me to work at home comfortably	3	13	23	36	26
Usefulness of distance learning	Evaluation of the success in distance learning is quite objective	8	13	33	44	3
	Distance learning provides me with a valuable learning experience	3	8	33	51	5
	A degree in distance learning is as valuable as a degree in traditional education	15	21	33	18	13
	I believe distance learning is useful	3	15	31	31	21
	Distance learning minimizes the inequalities in education	15	15	46	21	3
Distance learning satisfaction	In this course, I am pleased with the timely responses to my questions	0	5	18	44	33
	The content of this course meets my expectations	0	3	33	59	5
	I advise other students to take this course	0	0	23	67	10
	I like the content of the course which draws examples from real life	0	3	18	59	21
	The student-centred instruction offered in this course through distance learning is enjoyable	0	15	36	46	3

*SD, Strongly Disagree; D, Disagree; N, Neutral; A, Agree; SA, Strongly Agree*.

With the pandemic, telehealth has become an essential need for the general populace. Health care providers, and patients with COVID-19, especially when patients are in quarantine, telemedicine has enabled patients to contact the health care provider in real-time for advice on their health issues (84–86). Several technologies have been deployed for telehealth, including mHealth (mobile health), video and audio technologies, digital photography, remote patient monitoring (RPM), and store and forward technologies. In fact, one needs to replicate the entire health system in the “microcosm” of telehealth through information technology (87). Clinicians are an essential part of this microcosm, where they are involved in efficient management of patients and eliminating gaps in knowledge. Additionally, telehealth allows increased satisfaction of delivering superior patient care, and also grants decreased risk of burnout by providing a more manageable workload and improved patient communication processes. In line, it is imperative that medical education integrates aspects, whereby computer expertise of future clinicians are augmented and they are suitably prepared to address needs of patients through telehealth. Computer skills are essential for the effective implementation of telehealth strategies (88). As our DL learning framework strengthens and enhances computer expertise (Table 2), it demonstrates that it will allow medical students to be better prepared to integrate the precepts of telehealth in their clinical practice as well as during their clerkship years.

Furthermore, respondents' rate of 64% within the range of agree-strongly agree was recorded in the present study for the item regarding efficiency in internet usage gained through the online DL experience (Table 2). Also, 46% of respondents agreed that course assignments and projects augmented their computer skills (Table 2). The H&N course employed rubric-based assessments tailored employing Miller's model of competency. Hence, it can be safely concluded that incorporation of computer and internet as study tools in this course assisted students to improve their internet-searching/browsing skills, as well as develop their abilities to think critically and evaluate the credibility of the information sourced. Again, we believe this has long-term ramifications. Bhatia et al. showed that providing training for improvement of internet-searching skills for obtaining up-to-date medical information, and evidence-based medicine from the internet improves the practice of medicine (89); based on which one can conclude that our DL framework trains students in the conscientious, explicit, and judicious use of the current best evidence to inform decisions about the care of individual patients.

The last item in category 1 also yielded a significant response of 21% for the strongly disagree-disagree continuum (Table 2). Factoring in the aspect that the H&N course was delivered solely through DL, it involved considerable self-directed learning, which is based on the premise that learners are capable of determining their personal learning needs and set appropriate steps to achieve their personal learning goals (90). In contrast, in face-to-face delivery of a course, instructors often set the task for their students who are metacognitively, motivationally, and behaviourally active participants in their own learning process (91). The H&N course is delivered in the 1st year of the medical curriculum, and at this stage students may lack the academic maturity to address their learning needs entirely through self-directed learning [reflected in the significant disagreement observed (Table 2)]. The integration of blended sessions (92), may address this aspect, and therefore requires future investigation.

One word of caution, as the framework integrates the use of social media application (for the discussion of relevant clinical cases) (Figure 3) and the internet, it is imperative that students are suitably informed regarding the ethical implications that surround internet use and patient data confidentiality (93). To address this aspect, students who registered for the H&N course had to complete two courses in semester – 1 (Figure 1): A. Principles of Bioethics and B. Foundation in Clinical Medicine, which informed them about ethical considerations regarding medical practice and patient data confidentiality.

#### Flexibility of Distance Learning

In light of productive learning experiences, the concept of flexibility and its influence on three key dimensions of DL (i.e., learner engagement with the learning environment, learner-content engagement and learning experience design) were investigated through four items in category 2 i.e., flexibility of DL.

Item 1, which was reflective of the dimension “*learning experience design*,” addressed the effect of DL on students' time management skills (Table 2). Respondent rates for agree-strongly agree and strongly disagree-disagree continua were 46 and 36%, respectively (Table 2), with the former owing to the realistically achievable learning objectives outlined in the ADDIE model of instructional delivery (Figure 3). Since the ADDIE model is best known for its flexibility and cyclic nature (94), the well-structured content and organized workload may have enabled students to direct focused protected time to their fundamental learning needs. Moreover, the integration of both asynchronous and synchronous DL (95) components in the instructional delivery may have fostered students' self-regulation in multitasking behaviors. However, despite its specificity and flexibility, the ADDIE model is also scalable (96), and may necessitate that the individual dedicates more time to particular steps and actions. This may account for the 36% of respondents who were unable to allocate their time accordingly (Table 2).

Items 2 and 3, which were indicative of the DL dimension “*Learner engagement with learning environment*,” enquired about the conduciveness of the student's learning environment, and the flexibility of the DL modality in terms of time and location, respectively. With the number of COVID-19 cases rising exponentially at the commencement of the lockdown, social distancing measures became the most effective means of prevention (97). Hence, it is not surprising that the majority of respondents (62%) (Table 2), felt psychologically safe and physically comfortable to learn via the online DL platform during this time. Yet, DL may not prove advantageous for every student due to the apprehension associated with the use of technology and the physical separation between the instructor and student (98). It is also a possibility that collaborative learning and networking suffer at the expense of DL, leading to student frustration, uneasiness and confusion—all of which may impede the learning process (99). This may provide a plausible justification for 16% of respondent rates which were localized within the scale of strongly disagree and disagree (Table 2).

Despite the dispersion of responses across the strongly disagree-disagree and strongly agree-agree ranges for the conduciveness of the DL environment, 87% of students expressed a neutral response regarding DL flexibility in terms of time and location (Table 2). In fact, Dhawan stated that the flexibility of time and location in DL may be considered to be both strengths and weaknesses, the excessiveness of which may be beneficial or problematic (99). In this study, the preponderance toward this neutral opinion may imply that students were not comfortable to express themselves acquiescently. The rationale behind this selection may be explicated from the perspectives of the institutional DL platform and the home learning environment. In addition to the integration of the ADDIE model, the online delivery of the H&N course also adhered to the institutional teaching schedule using the WISE scheduling module (*Refer to methods for details*). While schedule adherence ensured that all required concepts were delivered timeously, it may have compromised the flexibility and time allocation of the DL modality.

It is notable that the predictability of the course schedule forms a pivotal component of the online syllabus in DL, as it instills routine, assists students to transition from one lesson to another, and encourages student productivity and enhanced learning (100, 101). Given the immense pressure faced in the transition to the DL modality, the online course schedule also allows instructors to distribute their time between teaching and scholarly duties, with sufficient time devoted to the design, development and implementation of online instructional approaches (102). However, the integration of more asynchronous learning components may present as a potential future improvement that paves the way for further flexibility in the DL framework.

Of course, the home learning environment also plays a role in the student's academic and social development (103). For many families, the mandatory shift to the home environment was viewed as a dynamic process, rather than an event because it entailed adjusting to a common workspace, thus requiring individuals of especially larger families to occupy designated workstations within the confines of their homes. It also meant that these individuals had to simultaneously develop a degree of tolerance and build a mental forbearance to overcome distractions. With quarantine becoming a normal part of life, boundaries of time and location were eventually invisible as individuals diverted their focus to achieving study goals and work deadlines, all at the cost of unbalanced circadian rhythms and mental well-being. In some communities, female students experienced challenges to juggle household chores and their studies, leading to poor academic performance and low morale (104). This highlights the cultural and societal disparities with which DL must contend. Indeed, respecting personal boundaries and priorities may allow families to work and study efficiently from the home environment, but motivating one and another to adhere to a daily routine may be the answer to mimic a typical day outside of quarantine.

The DL dimension “learning-content engagement,” which described how the student applied his/her learning style to engage with the H&N course content, was represented by item 4 in category 2. Respondent rates regarding the appropriateness of the DL modality to students' learning capacity were relatively similar for the strongly disagree-disagree (36%) and agree-strongly agree (41%) continua (Table 2). This observation alludes to several aspects, which we believe warrants further investigation.

Firstly, this observed dichotomy may be because of different learning methods availed by medical students. Visual, auditory, read, and kinetics (VARK) learning styles are adopted by students, but majority have been found to embrace the aural style (105). Therefore, it may be a possibility that the dichotomous distribution of responses alludes to contrasting learning styles present in the student cohort.

Secondly, the educational veracity of the medical curriculum is acutely susceptible to the effects of COVID-19. An early emphasis on clinical teaching has been an essential element of medical education reform in recent years (106). Medical curricula, including ours, now observe to abide by a strict guide-plan: an abridged preclinical period where students are educated within the medical school, and a subsequent clinical component during which students operate externally to their medical school and within the healthcare environment (107). This shift in pedagogy necessitates that preclinical students convene in groups for tutorials, problem-based learning, anatomy laboratory sessions and simulated patient interactions and for that clinical students have to access patient care centers. Although lecture-based teaching is easily transitioned to an online format, interactive small-group sessions and clinical exposure are not as easily replicated [*which we addressed through the integration of clinical case discussions and incorporation of WhatsApp discussion groups* (Figure 3)]. Given these reforms to curricular structure through the integration of the DL framework, the COVID-19 pandemic may have birthed an exasperating dichotomy for medical students (Table 2). A virus that exploits human contact for survival is encumbering an educational ecosystem that also necessitates human interaction.

Lastly, in designing the framework we employed the ADDIE model (Figure 3). ADDIE uses a behavioral approach in designing instruction (108). Behaviorism, cognitivism, and constructivism are the three primary learning theories (109). In behaviorism, information is transferred from instructors to learners from a response to the right stimulus. Students are a passive participant in behavioral learning— “instructors are giving them the information as an element of stimulus-response” e.g., through the discussion of a clinical case where the instructors elaborate to the students how the information that is gained through the didactic sessions is applied in addressing specific questions related to the clinical case. Instructors use behaviorism to show students how they should react and respond to certain stimuli. This needs to be done in a repetitive way to regularly remind students what behavior an instructor is looking for, as in the current study it was the through the repetitive discussion of relevant clinical cases in the WhatsApp group (Figure 3). Therefore, through ADDIE, although we were able to achieve the desirable outcomes in a DL learning environment, it also restricted us from applying reflective thinking (cognitivism), such as that which is done during active dissection sessions in the anatomy laboratory; and reflection in action (constructivism), which happens through collaborative construction of knowledge during group discussions following an active dissection session. The above may be responsible for the observed dichotomy of responses (Table 2). A blended approach may address these issues, but during the mandated lockdown integrating such sessions in the DL framework were not possible.

#### Usefulness of Distance Learning

Category 3 investigated the usefulness of the DL modality through five items. For items 1 (52%) and 3 (56%), students agreed that the DL framework was useful in the delivery of the H&N course, providing them with a valuable learning experience (Table 2). This is most likely because the DL framework allowed students to manage their time, better enabling them to effectively address the learning outcomes of multiple courses. Also, the DL framework employed the ADDIE model, which was disseminated using a published social media application interactome, that embodied aspects of peer-assisted learning and social constructivism (25). Thus, use of this instructional framework promoted and ensured active student participation and engagement, as observed in other similar studies (96).

On the contrary, as the H&N course was the inaugural structure-function course to be delivered via DL, students' responses within the strongly disagree-disagree continuum for items 1 and 3 (Table 2), may have stemmed from the questionable development of psychomotor skills in the absence of practical sessions.

Students' learning style is also considered to play an integral role in the usefulness and value of a DL ecosystem. As a result, learning style was recognized to be a possible inequality in education and was addressed through item 4. Although Mohr et al. argued that online DL enables students to capitalize on all four stages of Kolb's learning styles and experiential learning cycle (110), only 24% of respondents agreed that online DL abates such inequalities in education, with nearly 50% of respondents expressing a neutral opinion, which implied uneasiness and reluctance of students to share their views Table 2) (111). The latter may be justified by the ambiguity of the phrase “inequalities in education” as students may have misinterpreted it as access to online resources or components of social inequality.

With the establishment of massive open online courses (MOOCs) on the rise, obtaining a degree through distance education has gained considerable popularity as it promotes lifelong learning by extending beyond the boundaries of age, time and geographic location. Two types—Connectivistic Massive Open Online Course (cMOOC) and Extended Massive Open Online Course (xMOOC) have distinctly emerged. cMOOC promotes creativity and interaction among participants, while xMOOC is used merely for knowledge dispersion (112). However, the usual societal belief that on-campus degrees are more accepted and respected than those obtained through DL/MOOCs, tend to influence one's willingness to enroll in the latter (113). In the same vein, item 2 enquired about the comparable value of obtaining a degree through the traditional route vs. DL. The responses to this question were met with much skepticism as they were almost equally distributed across the strongly disagree-disagree, neutral and agree-strongly agree continua (Table 2). It is inevitable that the diversity of these responses hint at a multifactorial origin. Respondents' neutral opinion may be linked to the uncertainty of institutional reputation, employability, and duration of program. Further to the substantially high internet usage in the UAE, the recent multinational study of Fidalgo et al. reported that the inability of UAE undergraduate students to appraise the credibility of online DL programs was attributed to the lack of accreditation provided by the UAE Ministry of Education (114–117). Feelings of isolation, missing out on campus life, financial uncertainties, capricious internet connectivity, lack of immediate feedback and the communication gap between instructor and student may explain the negative stigma attached to obtaining a degree through DL (117). Given that medicine is a profession that entails the practice of hands-on skills, studying it through DL may have been viewed as a disadvantage to respondents. On the other hand, respondents' levels of agreement (Table 2), were most likely swayed by the global availability of numerous course and program offerings, autonomy to work independently and manage class- and study-time, opportunity to develop leadership skills, cost-effectiveness of the program and the eradication of traveling (118).

Of course, the success of DL also depends on the delivery and outcome of student assessment, and not merely on the delivery of instruction as it provides the opportunity to improve the modality. In item 5 of this study, 47% of respondents concurred that the success of DL in the H&N course was objectively evaluated (Table 2). The timely online delivery of both formative and summative proctored assessments through the ExamSoft platform in this course, as well as review of the item analysis by the course team, ensured that exam integrity was maintained. Through participation in formative assessment, students also had the opportunity to familiarize themselves with the assessment software and were provided with step-by-step troubleshooting guidelines in the event of technical hurdles.

#### Distance Learning Satisfaction

Students' satisfaction regarding online DL in the H&N course was explored in entirety through five question items that focused on enjoyment of student-centric instruction, expectations met by content, applicability of real-life examples through the discussion of relevant clinical cases (exemplar shown in Figure 3), course recommendation to other students, and provision of timely feedback disseminated using a previously applied strategy (7). Respondent rates of 49–80% were recorded within the agree-strongly agree continuum for all five items (Table 2), indicating that the DL delivery mode of this course was positively received by the students. This may be because of the application of pedagogical theories of Harasim's online collaborative learning, Garrison's community inquiry and Siemen's connectivism for blueprinting the DL framework and the subsequent integration of the ADDIE model of instructional design for the dissemination of the framework (Figures 2, 3). Interestingly, while the students in this study were exposed to dissection in previous structure-function courses, 80% of respondents agreed that the content of the course drew from examples of real-life (Table 2), proving that the course team succeeded in supplementing aspects of clinical and living anatomy through incorporation of a pedagogical framework in DL.

#### Preferred Mode of Distance Learning Delivery

In this study, the order of respondents' preferred mode of instructional DL delivery was noted to be hybrid/blended (i.e., combination of online and face-to-face instruction) (72%), on-site face-to-face (56%) and online/internet (26%) classes (data not shown). The preference of hybrid classes alluded to the existence of diverse learning styles and capacities within the respondent group and may suggest a potential recommendation for future direction. In fact, Hoch and Dougher documented an increasing trend of preference for hybrid classes over the years that was correlated to improved student attitude and overall class satisfaction (119). According to Marquis and Ghosh, hybrid classes are more attractive to the current generation of students as the flexibility enables them to maintain work-life balance and gain a greater sense of community (120).

### Kirkpatrick's Level 2: Cognitive Development

**Figure 6:**
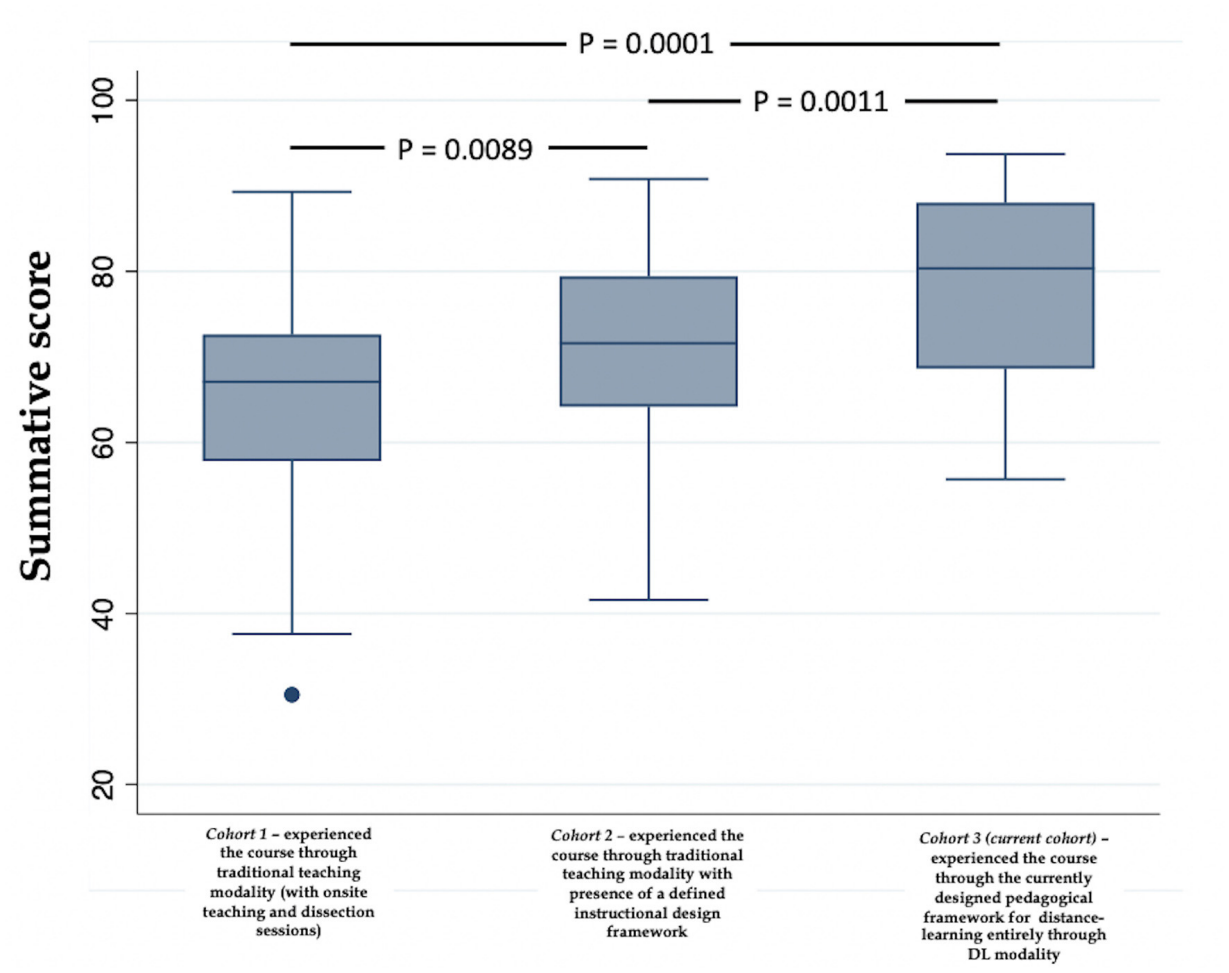
Comparison of the summative assessment performance in the H&N course across three cohort of students using ANOVA. Note: The implementation of a defined instructional design model along with a defined pedagogical framework (cohort−3) beneficially affects students' performance.

For cognitive development we compared the summative assessment performance in the H&N course across three cohort of students *(refer to methods for details on the different cohorts*) using ANOVA (Figure 6). The summative assessment was designed according to the principles of Miller's pyramid of professional competence (68), and employed different types of assessment tools (Figure 4) As observed in Figure 5, the median of summative assessments across three cohorts do not overlap with one another, highlighting that the presence of an instructional design model and/or pedagogical strategy has an effect on student performance in summative assessment i.e., cognitive development, where the presence of both instructional design model and pedagogical strategy, as is the case for the current cohort, which experienced the course through the DL framework, advantageously affects cognitive growth (*refer to the median of cohort*−*3 which has the highest value*). A *P* < 0.05 was observed when the mean of summative assessment was compared between: cohort−1 vs. cohort – 2; cohort – 2 vs cohort – 3; and cohort – 1 vs cohort – 3 (values are indicated in Figure 6) indicating statistical significance.

In summary, the implementation of a defined instructional design model along with a defined pedagogical framework augments cognitive growth. This observation is not unique to this study. Ogrnic et al. showed that pedagogical frameworks integrating practice-based learning, improved learning in medical students and residents ultimately augmenting patient care (121). Ross and his colleagues designed a pedagogical framework focusing on four domains of teaching: “Facilitating,” “Managing,” and “Learning and Community Building.” Students found the designed framework of learning and teaching not only helpful, but felt it adequately represented the place of teaching activities within the wider context of undergraduate medical education (122). Similarly, integration of instructional design models in course dissemination have also been shown to be advantageous for student learning (123). 25.

## Discussion

In this study, we designed a DL-framework, employing the DL theories of Garrison's community inquiry (36), Siemens' connectivism (38), Harasim's online-collaborative-learning (39); and Anderson's DL-model (40). The blueprinted framework was successfully used in the delivery of H&N anatomy course in the pre-clerkship phase of the competency based medical curriculum at MBRU. In order, for course dissemination we integrated the ADDIE instructional model in the framework (41). The efficiency of the framework was evaluated using first two levels of Kirkpatrick's framework (42).

Although in the literature there are numerous exemplars and instances of DL modes of course delivery specifically relating to course delivery during the pandemic (124–128); to our knowledge this is the first-study of its kind where a rationale and theory-guided approach has been availed not only to blueprint the framework, but also to implement it in the undergraduate medical curriculum.

Case in point, the study by Maggipinto et al. although provides the readers with valuable video resources that can be effectively used to deliver courses in medical genetics in the pre-clerkship phase of the curriculum, but unlike our study, falls short on informing the readers regarding how these video resources can be used to create a framework to effectively deliver a course in medical genetics through DL modality (128). Hence, it is safe to conclude that the outcome of the present study is a DL-framework that is highly versatile and robust; and although in the present study we have employed the framework to deliver a course in anatomy, the designed framework can be easily adapted for the delivery of any course in undergraduate medical curricula with minor tweaks. In fact, in a recent study by our research group, precepts of the framework were adopted with minor modifications in delivering a course in epidemiology and biostatistics. Readers are referred to the article by Azar et al. for details (61).

**Figure 7:**
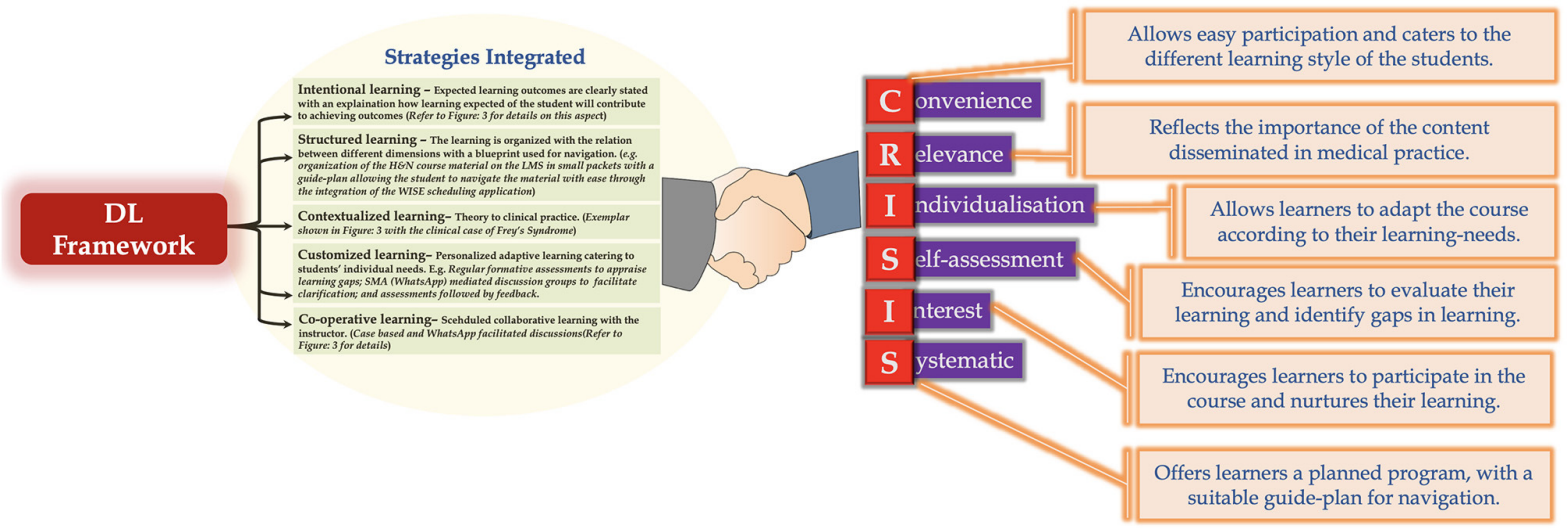
Strategies integrated in DL framework and how they align with CRISIS framework of Harden. Note: All learner-centric strategies could be integrated in the DL framework except blended learning.

Robustness of our DL-framework is explicated by the fact that it integrates diverse student-centred educational strategies (Figure 7). Unfortunately, because of the mandated COVID-19 lockdown in place, we couldn't integrate aspects of “blended learning” in our DL-framework (129), which would have further augmented the framework's cogency. In line, it is warranted that practitioners of health professions education who are interested in using/adopting this framework for course delivery should also incorporate principles of “blended learning” into the framework which we believe would further augment the benefits of the framework.

The integration of student-centred educational strategies allows our DL-framework to attest to the CRISIS model of Harden (Figure 7) (130). The CRISIS model is widely used in medical education and has been extensively employed in designing and delivering continuing medical education activities at the University of Dundee (131). The model has also been utilized in course design and delivery, in areas other than medicine, which attests to CRISIS' versatility and ease of adoption according to the need of the situation. For example, Dunn & Hamilton applied a modified version of CRISIS to an assessment of continuing education for pharmacists (132). Additionally, the rationale of the CRISIS model has also been investigated, and is related to Brookfield's six principles of effective practice (133), in harbingering adult learning (134). The fact that our DL-framework attests to the CRISIS model further highlights its robustness and easy adaptability.

**Figure 8:**
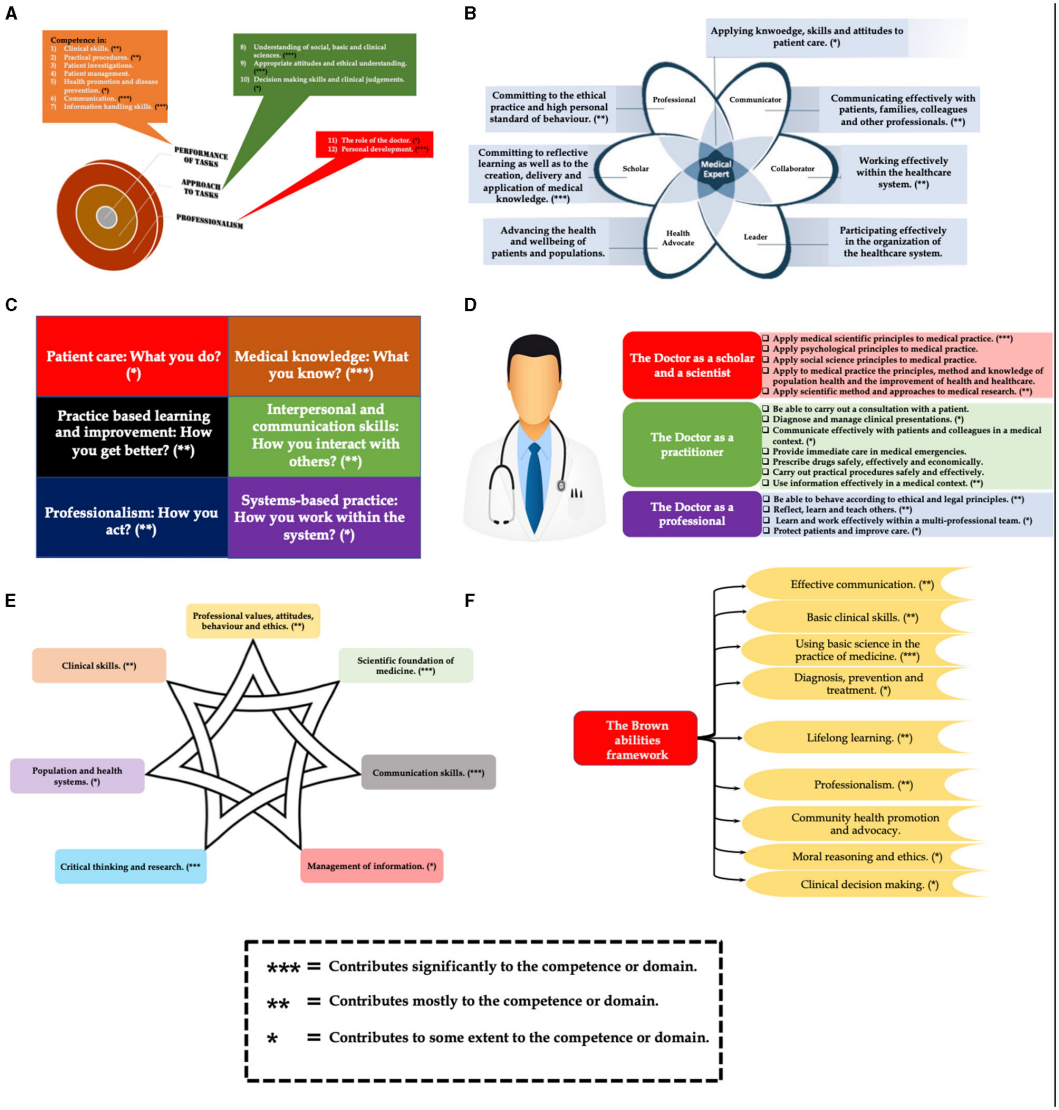
Alignment of the designed DL framework with several of the key domains of: **(A)** the Scottish Doctor framework; **(B)** the CanMEDS physician competency framework; **(C)** the Accreditation Council for Graduate Medical Education (ACGME) competency framework; **(D)** the General Medical Council (GMC) UK competency framework; **(E)** the Global Minimum Essential Requirements (GMER) competency framework; and **(F)** the Brown abilities competency framework.

Furthermore, the ingenuity and versatility of our DL-framework is demonstrated by the fact that it endorses several of the key domains of the different learning outcomes framework (Figure 8). As shown in Figure 8, the designed framework aligns well with several of the key domains of: the Scottish Doctor framework (Figure 8A) (135); the CanMEDS physician competency framework (Figure 8B) (136); the Accreditation Council for Graduate Medical Education (ACGME) competency framework (Figure 8C) (137); the General Medical Council (GMC) UK competency framework (Figure 8D) (138); the Global Minimum Essential Requirements (GMER) competency framework (Figure 8E) (139); and the Brown abilities competency framework (Figure 8F) (140).

**Figure 9:**
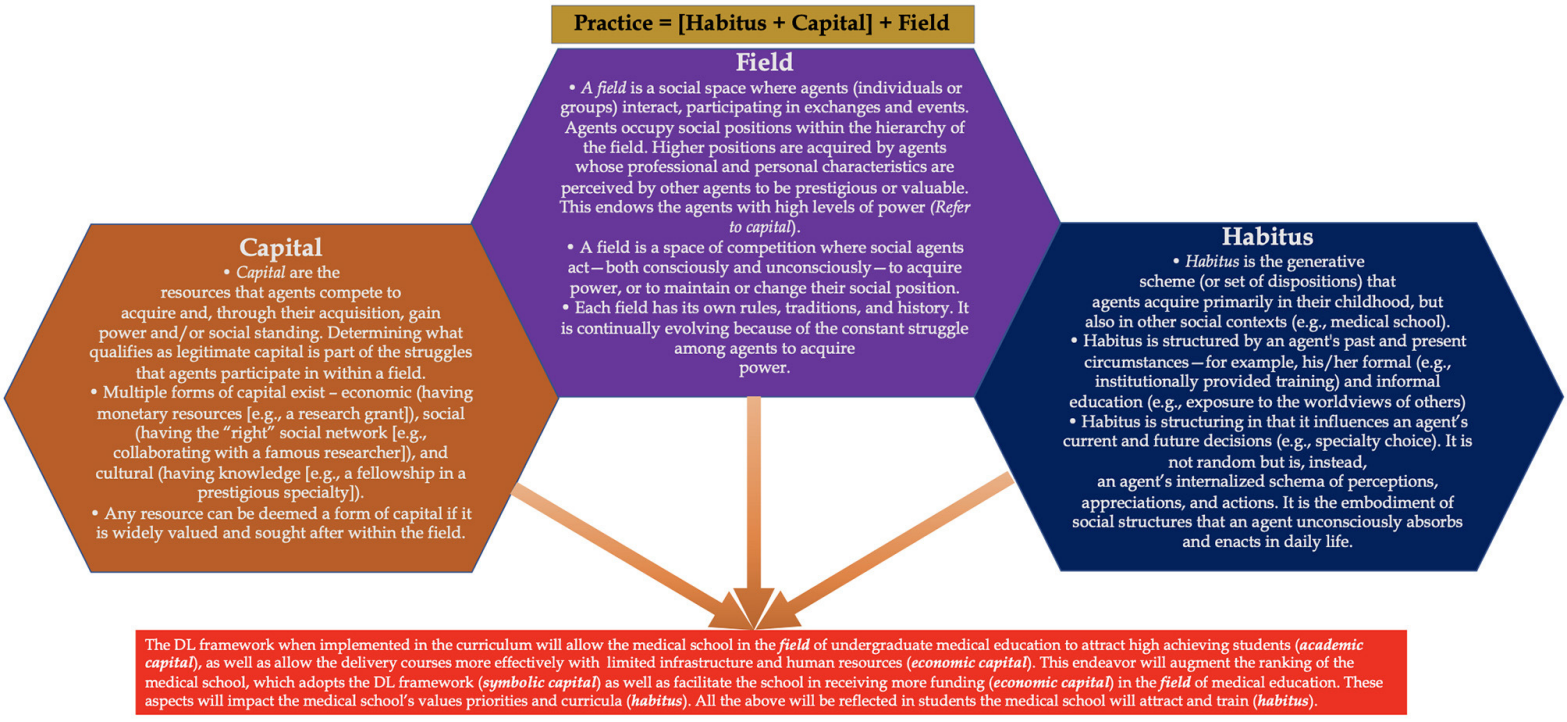
Bourdieu's Theory of Practice. The figure elaborates on three intimately related concepts: field, capital, and habitus. The text box in RED elaborates how Bourdieu's Theory of Practice when applied to the current context demonstrates the benefit of the DL framework being adopted by a medical school.

Based on the above observations, the DL-framework presents an excellent pedagogical approach, which, when adopted by any medical school, will have long-term plusses and advantages. This aspect is important as a recent study indicated that temperature and latitude do not appear to be associated with the spread of COVID-19, instead school closures and other public health measures seem to have a positive effect (141, 142). Also, significant degree of vaccine hesitancy observed in medical students (143). These reflect that it is pertinent that medical schools avail DL modality to address students' learning needs, indicating the need for a robust and versatile DL-framework. This can be further elaborated using Pierre Bourdieu's Theory of Practice (144–146). Bourdieu developed three intimately related concepts: field, capital, habitus (Refer to Figure 9 for details of the individual concepts). By applying Bourdieu's Theory of Practice, the designed framework, when executed and integrated in a competency-based medical curriculum, will allow any medical school to function effectively to deliver medical education, even during unprecedented times as presented by the current COVID-19 pandemic, to attract high achieving students (*academic capital*), as well as allow a more effective delivery of courses with access to limited infrastructure and human resources (*economic capital*). This will augment the ranking of the medical school, which has adopted the DL-framework (*symbolic capital*), as well as facilitate the school in applying and receiving more funding or emoluments (*economic capital*) in the field of medical education and health professions education research. These aspects will impact the medical school's values, primacies and curricula (*habitus*). Furthermore, all the above will be reflected in students the medical school will attract and train (*habitus*).

Although our DL pedagogical framework has overarching and specific benefits (discussed extensively in RESULTS and DISCUSSION sections), there are specific limitations that need to be addressed:

1. In this study we have evaluated only the first two levels of Kirkpatrick's framework. However, levels – 3 and 4 of the framework, corresponding to “Did the intervention bring about a change in behavior?” and “Did the intervention influence performance?” respectively, still need to be evaluated. To pursue these evaluations, long-term studies are warranted, where the DL framework needs to be adopted across courses in both pre-clerkship and clerkship phases of curriculum, following which the effect of this intervention has to be assessed using suitable tools. For assessment of level – 3 of Kirkpatrick's framework, behavioral analysis of the ward rounds of students, exposed to the DL framework across different courses in the curriculum, is required. In this regard, the methodology of Sanson-Fisher et al. can be employed (147). Evaluation of level – 4 can be pursued using a strategy analogous to Seeley and Harding (148). Here, one group of students (the experimental group) will be exposed to the DL pedagogical approach in different courses in the curriculum. A second group of students (the control group) will attend courses delivered using traditional teaching methods. Dedicated multiple-choice question assessments and objective structured clinical examinations (OSCE) will be used to evaluate knowledge and skills. Results will indicate if the experimental group shows improved post-intervention clinical practice compared with the control group.
2. One of the key limitations of this study is the small sample size. The small size of the sample is justified by the following factors: (A) As per the guidelines of the Commission of Academic Accreditation (CAA) in the United Arab Emirates (UAE), and given that MBRU is a newly established institution, it is limited to an intake of 70 students per year until the first cohort of students has graduated, which will be in 2022; (B) In the transition from semester 1 to semester 2 of year 1, there is notable attrition (~20 students) in the year 1 cohort, with ~50 first-year students successfully progressing from semester 1 to semester 2; and (C) In light of the diverse high school curricula in the UAE, many hopeful high school students may not satisfy the basic entry requirements outlined by MBRU, resulting in the receipt of fewer applications.
  The authors also attempted to detect a simple correlation between DL modality and academic performance using a 2-sided test of 5% significance level (alpha = 0.05) with 80% power (β = 0.20) with correlation coefficient *r* = 0.20 of N observations. The required sample size was then estimated to be 194 (Table 3). However, as per the mandated guidelines of the CAA, a sample size of this magnitude is not possible. Consequently, this shortcoming will be addressed using a multi-centric approach in the future, where the DL strategy is applied to disseminate content pertaining to the regional anatomy of the H&N across different medical schools in the UAE, thereby establishing one of the most important goals of our future investigations.
3. Anatomy as a discipline in a typical competency based undergraduate medical curricula, necessitates that the students are exposed to dedicated hands on sessions either through the use cadaveric dissection (149), or through the dedicated utilization of virtual reality modules (150). In other words, the dissemination of anatomy courses require the judicious use of manipulative activities especially dissection sessions. Manipulatives are concrete or virtual objects generally used in the elementary mathematics curriculum to disseminate fundamental concepts, thereby promoting hands-on learning. The major use of manipulatives in college education has been in chemistry classes, where students learned to construct molecules with model kits. Their use in medical education has been so far limited. Case in point, Krontiris-Litowitz employed manipulatives to enhance learning of neurophysiology in the medical curriculum (151).
  Manipulative activities augment student-engagement. Such activities also build on a concept, taking it from the simplistic to the complex, thus facilitating horizontal and vertical integration. Additionally, manipulative activities address different learning styles, addressing the needs of visual, auditory, and kinaesthetic learners (152). In our study, we integrated manipulatives through a two-pronged approach which consisted of the use of: (A) 3D visualization module (BioDigital Interactive 3D Anatomy application), and (B) alternative assessments (Formative quizzes comprising of open-ended questions). An exemplar of such a quiz where an instructor discussed a long-case based on the case report of Williams et al. (153) is shown below:
  “*A 5-year-old girl presented to her GP with a history of an erythematous rash that appeared on her left cheek associated with eating certain foods including strawberries, apples, and sweets. The rash would appear immediately on mastication and would entirely disappear within 30 min of ingestion*.
  *Her medical history was unremarkable apart from a road traffic accident at 3 years of age when she suffered facial and chest trauma leading to a mandibular fracture and right lower lobe collapse. The patient required intubation and ventilation for 9 days on the pediatric intensive care unit and underwent maxillofacial surgery for the mandibular fracture*.
  *Physical examination revealed a well-grown child with no systemic abnormalities or eczema. Within a few seconds of eating candy a facial flushing appeared on her left cheek, stretching from her the temporal region to the corner of her mouth. This faded within a few minutes*.
  *(i) Based on the above clinical presentation what is the most likely diagnosis that the GP will make?*
  *(ii) What is the differential diagnosis for this patient?*”
  Such formative quizzes were discussed in detail via the social interactome model (more specially in the WhatsApp groups) (Figure 3). To stimulate discussion in the groups, an array of probes in line with those identified and typified by Brown and Atkins (154) were employed. The key aim of such discussions were to address the fact that when making a diagnosis of Frey's syndrome one should keep an open mind.
  As our DL-framework was specifically tailored for the delivery of courses during the mandated lockdown period associated with COVID-19 pandemic, manipulative activities in the form of hands-on or dissection sessions couldn't be integrated into the framework. Although, we have tried to assuage this shortcoming through the integration of the two-pronged approach presented above, which was positively received by students; we perceive that students may feel less confident to pursue specific tasks specifically during their surgical rotation especially in the operating room. Cadaveric dissection integrated in anatomy courses aim to close the gap between the anatomic knowledge and surgical practice. Students gain knowledge and strengthen theoretical data through visualization of real anatomic structures. Additionally, by practicing on cadavers they touch and feel the anatomic relations more efficiently (155). This aspect requires further investigation.
4. Although our DL-framework has been able to provide an enriched learning experience to students in the H&N course, will it be able to do the same across all the courses in the pre-clerkship phase of the curriculum?, specifically in those courses where there is a relative dearth of clinical scenarios/cases/vignettes. such as courses associated with research methodology and ethics. To address this issue, we are in the process of implementing precepts of the DL-framework in the delivery of courses such as Research Methods and Principle of Bioethics. Results from these studies are awaited and will form the basis of future scholarly communications.
  One of the reasons we were able to successfully implement our teaching approach within a limited frame of time can be attributed to the presence of well-structured e-learning and cyber resources at our university, which we have alluded to, in the methodology. However, “Can our framework be adopted effectively by medical schools with limited access to such resources?,” is a question that still needs to be addressed especially for medical schools in developing countries (156). One of the cost-effective strategies for medical schools with limited access to e-learning and cyber resources, will be to implement social media applications (SMA) such as YouTube channels and WhatsApp discussion groups (*which we have also integrated into the framework*) in the delivery of courses through DL modality. In fact, our previous study indicates that these two SMA are regularly used by medical students in their learning process (46). Additionally, instructors with limited access to e-learning resources can employ virtual classroom modules such as WizIQ (https://www.wiziq.com) (104), which provide flexibility of pricing.
  Lastly, it needs to be assessed if our teaching framework will be effective in the delivery of courses involving patient exposure. One of the ways to address this aspect will be to integrate the principles of telemedicine in our framework. A fundamental strategy for healthcare surge control is “forward triage” or the sorting of patients prior to their arrival in the emergency department (84). Direct-to-consumer (or on-demand) telemedicine, a 21st-century tactic to forward triage that permits patients to be competently screened, is equally patient-centred and advantageous to self-quarantine, and it safeguards patients, clinicians, medical students and the community from exposure. It allows physicians and patients to connect 24/7 using smart devices. Respiratory symptoms, which may be initial signs of COVID-19 infection are among the complaints generally appraised with this approach. Health care providers can effortlessly obtain complete travel and exposure histories. Automated screening algorithms are usually built into the intake process, and local epidemiologic information can be used to standardize screening and practice patterns across providers. In line, if precepts of telemedicine are integrated into our framework, especially in our virtual live online sessions, students will be able to interact safely with patients, even during the COVID-19 pandemic. However, this requires further investigation.

**Table 3:** Power calculation table [Significance level (α) = 0.05; N is indicated by the italicized numerical in the table].

	**Sample correlation (r)**							
**Power of the study (1-β)**	0.2	0.3	0.4	0.5	0.6	0.7	0.8
	0.9	259	113	62	38	25	17	12
	0.8	194	85	47	29	19	13	10
	0.7	153	67	37	23	16	11	8
	0.6	122	54	30	19	13	10	7

*Note: for association of DL modality to academic performance, with a range of power values (0.6–0.9) (shaded region), indicates that the number of participants will be suitable for statistical correlations. In line with the table, if one wishes to detect a simple correlation between DL modality to academic performance, where correlation co-efficient (r) = 0.2 of N observations, then using a two sided test, of 5% significance level (α = 0.05) with power 80% power (β = 0.2), the required sample size is ~194 (n = 194)*.

## Conclusion

In conclusion, in this study we have designed a student-centric and versatile DL-framework integrating precepts of ADDIE instructional model. The framework was used for the delivery of H&N anatomy structure-function course, and was not only received positively by the students, but also contributed successfully to their cognitive development. One of our future courses of action, will be to implement this framework in the delivery of other courses in the pre-clerkship phase of the curriculum. To this effect, we have designed a change-management strategy using Mento's change-management model (157). The strategy aims to facilitate a change in pedagogy such that instructors integrate the precepts of the DL-framework in their course delivery. In fact, the change-management strategy has been successful as precepts of the framework were integrated by instructors in the delivery of fundamentals epidemiology and biostatistics course during the mandated COVID-19 lockdown period (61).

Additionally, one our future aims will be to fine-tune and revise the DL-framework by integrating aspects of resilience and stress-coping. With regards to the latter currently a study is in progress to determine the relationship between resilience, learning approaches, and stress-coping strategies and how they can collectively predict achievement in undergraduate medical students (158). Specifically, the indicated study addresses: What is the correlation between the psychoeducational variables; resilience, learning approaches, and stress-coping strategies? Can academic performance of undergraduate medical students be predicted through the construction of linear relationships between defined variables employing the principles of empirical modeling? (158). Fine-tuning of the DL-framework, using data obtained from this study will further enhance the adaptability of DL-framework better addressing students' learning trajectories during unprecedented times such as that created by the current COVID-19 pandemic.

## Data Availability Statement

The original contributions presented in the study are included in the article/supplementary material, further inquiries can be directed to the corresponding author/s.

## Ethics Statement

The studies involving human participants were reviewed and approved by Written consent was obtained from all individual respondents included in this study as the pursued survey was an essential part of the feedback process pertaining to the H&N course. The study was considered as a quality control project. Therefore, according to the policy and guidelines, of The Mohammed Bin Rashid University of Medicine and Health Sciences-Institutional Review board (MBRU-IRB), this project doesn't necessitate appraisal by MBRU-IRB or an exempt status. Further clarification can be obtained from the MBRU-IRB at irb@mbru.ac.ae. The study protocol is in line with the MBRU-IRB guidelines. The study spanned between January and September of 2020 during the mandated lockdown period because of the COVID−19 pandemic. The patients/participants provided their written informed consent to participate in this study.

## Author Contributions

NN was involved in the delivery of the H&N course in the semester−1 of Phase–I (in the pre-clerkship phase of the MBRU curriculum) referred to in this study and drafted significant sections of the manuscript. AA and AK were responsible for the statical analysis. MG and ML were responsible for data collection and curation. AA-A assisted in data analysis pertaining to the section pertaining to Kirkpatrick's Level−2 and YB as the director of Phase−1, designed the study in order to implement change in teaching in Phase–I of the curricula at MBRU during the COVID-19 mandated lockdown, drafted the final version of the manuscript along with NN, and oversaw the general organization and logistics of the study. All authors have read and approved the manuscript.

## Conflict of Interest

YB was the recipient of funding from Pfizer, Amgen, and the BlueNote community to conduct investigator initiated research (IIR) and medical education activities in the form of continuing professional development (CPD) and continued medical education (CME) activities. However, these funds haven't been used in the study depicted in the manuscript. The remaining authors declare that the research was conducted in the absence of any commercial or financial relationships that could be construed as a potential conflict of interest.

## Publisher's Note

All claims expressed in this article are solely those of the authors and do not necessarily represent those of their affiliated organizations, or those of the publisher, the editors and the reviewers. Any product that may be evaluated in this article, or claim that may be made by its manufacturer, is not guaranteed or endorsed by the publisher.

## Abbreviations

EPA: Entrustable Professional Activities
DL: Distance Learning
OSPE: Objectively Structured Practical Examination
OSCE: Objective Structured Clinical Examination
H&N: Head and Neck course
CBMC: Competency-Based Medical Curriculum
ADDIE, Analyse, Design, Develop, Implement: and Evaluate
MBRU, Mohammed Bin Rashid University of Medicine and Health Science or in Latin: Medicinae Baccalaureus Baccalaureus Chirurgiae
MBBS, Bachelor of Medicine: Bachelor of Surgery
LMS: Learning Management System
MS: Microsoft
KMO: Kaiser-Meyer-Olkin
MSA: Measure of Sample Adequacy
EFA: Exploratory Factor Analysis
ANOVA: Analysis of Variance
GWAS: Genome-Wide Analysis Study
RPM: Remote Patient Monitoring
MOOC: Massive Open Online Course
ACGME: Accreditation Council for Graduate Medical Education
GMC: General Medical Council
GMER: Global Minimum Essential Requirements
SPSS: Statistical Package For The Social Sciences
SD: Standard Deviation.
